# The Impact of Intraoperative Respiratory Patterns on Morbidity and Mortality in Patients with COPD Undergoing Elective Surgery

**DOI:** 10.3390/jcm14072438

**Published:** 2025-04-03

**Authors:** Mariya M. Shemetova, Levan B. Berikashvili, Mikhail Ya. Yadgarov, Elizaveta M. Korolenok, Ivan V. Kuznetsov, Alexey A. Yakovlev, Valery V. Likhvantsev

**Affiliations:** Department of Clinical Trials and Intelligent IT, Federal Research and Clinical Center of Intensive Care Medicine and Rehabilitology, 25 Petrovka Str., 107031 Moscow, Russia; mshemetova@mail.ru (M.M.S.); levan.berikashvili@mail.ru (L.B.B.); mikhail.yadgarov@mail.ru (M.Y.Y.); ekorolenok@fnkcrr.ru (E.M.K.); ikuznecov@fnkcrr.ru (I.V.K.); ayakovlev@fnkcrr.ru (A.A.Y.)

**Keywords:** anesthesia, COPD, intraoperative care, pulmonary ventilation, length of stay, mortality

## Abstract

**Background/Objectives**: Surgical procedures in chronic obstructive pulmonary disease (COPD) patients carry a high risk of postoperative respiratory failure, often causing the need for mechanical ventilation and prolonged intensive care unit (ICU) stays. Accompanying COPD with heart failure further increases the risk of complications. This study aimed to identify predictors of mortality, prolonged ICU and hospital stays, the need for mechanical ventilation, and vasoactive drug usage in ICU patients with moderate to severe COPD undergoing elective non-cardiac surgery. **Methods**: This retrospective cohort study analyzed eICU-CRD data, including adult patients with moderate to severe COPD admitted to the ICU from the operating room following elective non-cardiac surgery. Spearman’s correlation analysis was performed to assess associations between intraoperative ventilation parameters and ICU/hospital length of stay, postoperative laboratory parameters, and their perioperative dynamics. **Results**: This study included 680 patients (21% with severe COPD). Hospital and ICU mortality were 8.6% and 4.4%, respectively. Median ICU and hospital stays were 1.9 and 6.6 days, respectively. Intraoperative tidal volume, expired minute ventilation, positive end-expiratory pressure, mean airway pressure, peak inspiratory pressure, and compliance had no statistically significant association with mortality, postoperative mechanical ventilation, its duration, or the use of vasopressors/inotropes. Tidal volume correlated positively with changes in monocyte count (R = 0.611; *p* = 0.016), postoperative lymphocytes (R = 0.327; *p* = 0.017), and neutrophil count (R = 0.332; *p* = 0.02). Plateau pressure showed a strong positive association with the neutrophil-to-lymphocyte ratio (R = 0.708; *p* = 0.001). **Conclusions**: Intraoperative ventilation modes and parameters in COPD patients appear to have no significant impact on the outcomes or laboratory markers, except possibly for the neutrophil-to-lymphocyte ratio, although its elevation cause remains unclear.

## 1. Introduction

Chronic obstructive pulmonary disease (COPD) is one of the leading causes of mortality worldwide, as reported by the World Health Organization (WHO) [1]. Although COPD-related mortality rates have declined across all age groups worldwide, this trend is likely attributable to advances in medical care—particularly earlier diagnosis, improved pharmacological therapies, greater access to treatment, and the widespread implementation of smoking cessation programs. However, the total incidence of COPD has surged by 86% over the past 20 years, with a 30% rise in COPD-related deaths [2,3]. These increases may be attributed to demographic shifts toward older populations, improved survival of individuals with chronic diseases, and the expanded use of diagnostic tools such as spirometry and advanced imaging in routine clinical practice, which have minimized undiagnosed cases [2,3].

According to AL Wachami et al., the moderate stage is the most prevalent form of COPD, affecting 50.46% of patients globally [4]. However, surgical procedures in patients with COPD are associated with a significantly higher risk of morbidity and mortality. This risk is primarily attributed to the increased likelihood of postoperative respiratory failure, particularly in severe cases of COPD [5,6,7].

Although clinical guidelines are available for managing COPD, there is still a lack of robust evidence regarding key factors—such as COPD phenotypes, comorbidities, and intraoperative management strategies (including ventilation settings and anesthetic techniques)—that influence mortality, intensive care unit (ICU) and hospital length of stay, and the need for mechanical ventilation (MV) or vasoactive support in these patients [8,9].

The objective of this study was to evaluate the impact of intraoperative mechanical ventilation parameters on hospital outcomes and the trajectory of laboratory biomarkers.

## 2. Materials and Methods

### 2.1. Data Sources

The data were acquired from the eICU Collaborative Research Database (eICU-CRD), developed by Philips Healthcare in collaboration with the Massachusetts Institute of Technology’s Laboratory for Computational Physiology. The eICU-CRD contains comprehensive clinical data from 200,859 patient admissions across 335 intensive care units (ICUs) in 208 U.S. hospitals, collected during the period of 2014–2015 [10]. All patient data were fully anonymized, neglecting the need for local ethics committee approval. One of the authors successfully completed training modules “Human Research: Data or Specimens Only Research”, and “Conflicts of Interest,” and was granted access to the eICU database (certificate numbers: 56653575, 56653561, valid until 21 June 2026).

### 2.2. Selection Criteria

All adult patients with moderate to severe COPD who underwent elective non-cardiac surgery and were subsequently admitted to the ICU from the operating room were included in this retrospective cohort study. Exclusion criteria were as follows: (1) patients aged 90 years or older, and (2) those lacking data on hospital discharge status.

### 2.3. Data Extraction

Data extraction was performed using SQLite version 3.45.2 (https://www.sqlite.org/ (assessed on 15 May 2024)) and DBeaver software v. 24.2.4. The extracted parameters included (1) general patient information, namely, sex, age, body mass index (BMI), APACHE IV (Acute Physiology and Chronic Health Evaluation) score at admission, and COPD severity; (2) surgical interventions; (3) comorbidities; (4) laboratory parameters; (5) intraoperative ventilation parameters, and (6) hospitalization outcomes. Mechanical ventilation duration was calculated in whole days; any usage within a day was counted as one full day.

The laboratory parameters contained values from 3 time points, including (a) preoperative values, defined as 1 to 5 days before ICU admission, (b) postoperative—first evaluation in the ICU, and (c) the percentage change from preoperative to postoperative values. Intraoperative ventilation parameters included positive end-expiratory pressure (PEEP), tidal volume (TV), mean airway pressure (P_mean_), peak inspiratory pressure (P_peak_), expired minute ventilation (EMV), plateau pressure (P_plateau_), compliance, peak flow, continuous positive airway pressure (CPAP), and the fraction of inspired oxygen (FiO_2_).

### 2.4. Outcomes

The primary endpoint was the association between intraoperative ventilation parameters and mortality in both the hospital and ICU settings (irrespective of the length of hospital or ICU stay). Secondary endpoints included correlations between intraoperative ventilation parameters and length of stay (LoS) in the hospital and ICU, association with the MV requirement, its duration, administration of vasopressors/inotropes, and postoperative complications such as heart block and/or ventricular arrhythmia, heart failure, stroke, acute kidney injury, acute respiratory distress syndrome, respiratory failure, pleural effusion, congestive heart failure, sepsis, septic shock, thrombosis complications, and multiorgan failure.

### 2.5. Statistical Analysis

The distribution of the data was assessed using the Shapiro-Wilk test. Continuous variables were reported as medians (Mes) with interquartile ranges (IQRs), while categorical variables were presented as frequencies and percentages (%). The chi-square test and Fisher’s exact test were employed for comparing frequencies, while the Mann-Whitney U (Wilcoxon rank-sum) test was utilized to compare continuous variables. The grouping variable for comparing initial parameters and surgery types was the severity of COPD. The laboratory and respiratory parameters dynamics were assessed via a comparison of pre- and postoperative values (Wilcoxon signed-rank test). Spearman’s correlation analysis was performed to evaluate the association between intraoperative ventilatory parameters, postoperative laboratory parameters, and clinical outcomes, including correlation coefficient (R) estimation. Correlation strength (|R|) was categorized as negligible (0 to 0.3), low (0.3 to 0.5), moderate (0.5 to 0.7), high (0.7 to 0.9), or very high (0.9 to 1) [11]. A two-sided significance level was set at 0.05. All statistical analyses were performed using IBM SPSS Statistics v. 29.0 and Statistica 7.0 StatSoft, Inc. Visualization was performed using Python 3.11, including NumPy 1.26.0, Matplotlib 3.9.0, Seaborn 0.14.0, and Pandas 2.2.0 packages.

## 3. Results

**Figure 1:**
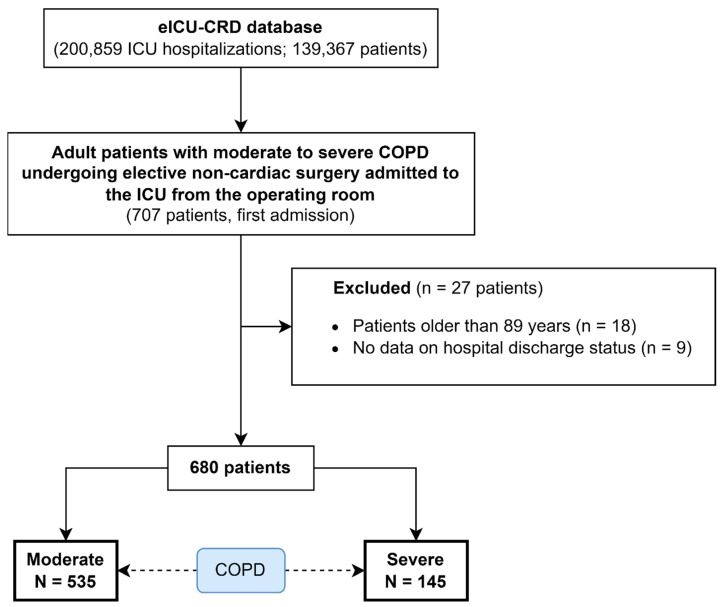
Flowchart of patient selection in the study.

The STROBE checklist for observational studies is provided in Supplemental S1. A flowchart illustrating the patient selection process is presented in Figure 1. A total of 27 patients were excluded sequentially according to the exclusion criteria.

A total of 680 patients were included in the analysis (361 males, 53%) (Table 1). The median age was 69 years (IQR 61 to 76) and the median body mass index (BMI) was 26.6 kg/m^2^ (IQR 22.5 to 32.5). The most frequent types of surgeries performed were abdominal (33%), thoracic (22%), and vascular (19%). Among the included patients, 61% had arterial hypertension, 12% had arrhythmias, and 11% experienced stroke. Chronic heart failure was present in 16% of the cohort, while 11% of the patients suffered from chronic kidney disease and 12% had insulin-dependent diabetes. Asthma was reported in 14% of patients, and 12% required home oxygen therapy. The percentage of patients with cancer was 17%. The median length of ICU stay was 1.9 days, and the median hospital stay was 6.6 days. Hospital and ICU mortality rates were 8.6% and 4.4%, respectively.

A statistically significant increase was established in postoperative glucose levels, white blood cell (WBC) count, neutrophil count, and neutrophil-to-lymphocyte ratio (NLR) with *p*-values < 0.001 (Table 2). Additionally, postoperative values elevated for total bilirubin (*p* = 0.002). On the contrary, there was a reduction in hemoglobin, hematocrit, albumin, total protein levels, red blood cells (RBCs), lymphocytes, and eosinophil counts with all the *p*-values < 0.001. Statistically significant decreases were also identified for platelet (*p* = 0.002) and basophil (*p* = 0.042) counts.

Intraoperative ventilation parameters such as TV, EMV, PEEP, P_mean_, P_peak_, and compliance had no statistically significant connection with mortality, MV or its duration, and vasopressor/inotrope use (Table 3).

The results of the correlation analysis between hospital and ICU LoS and ventilation parameters are presented in Table 4. There were no statistically significant associations except for the low positive correlation between hospital LoS and P_mean_, (R = 0.373; *p*-value = 0.008).

**Figure 2:**
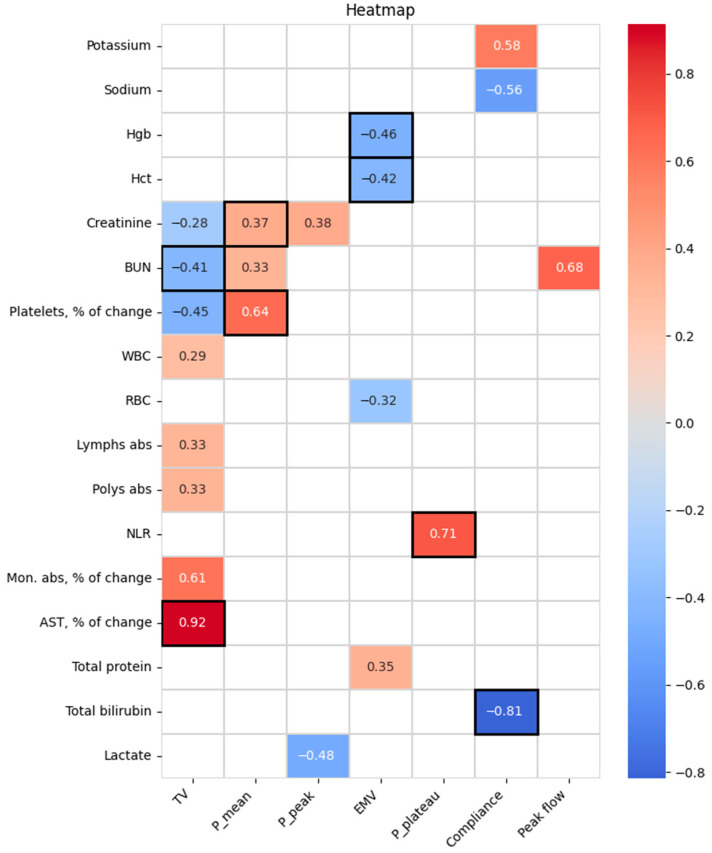
The heatmap for the statistically significant results of Spearman’s correlation analysis between intraoperative ventilation and postoperative laboratory parameters. Correlation coefficients (R) were presented if the *p*-value was <0.05 and the number of cases was ≥10. The associations with *p* < 0.01 are highlighted with black frames. Abbreviations: AST, aspartate aminotransferase; BUN, blood urea nitrogen; EMV, expired minute ventilation; Hct, hematocrit; Hgb, hemoglobin; Lymphs abs, absolute lymphocyte count; Mon. abs, absolute monocyte count; NLR, neutrophil-to-lymphocyte ratio; P_mean, mean airway pressure; P_peak, peak inspiratory pressure; P_plateau, plateau pressure; Polys abs, absolute neutrophil count; RBC, red blood cell count; TV, tidal volume.; WBC, white blood cell count.

The correlation analysis of associations between intraoperative ventilation parameters and postoperative laboratory parameters also revealed statistically significant associations as shown in Figure 2 and Table S2.

There was found a moderate positive correlation between TV and monocyte count change (R = 0.611; *p* = 0.016). TV also showed a low positive association with lymphocytes (R = 0.327; *p* = 0.017) and neutrophils (R = 0.332; *p* = 0.02) counts. However, the correlation of TV with WBC count was negligible (R = 0.286; *p* = 0.018), while the association between TV and NLR was not statistically significant. Among ventilation pressures, there was no correlation with immune parameters except for the high positive association between P_plateau_ and NLR (R = 0.708; *p* = 0.001). P_peak_ also showed a low negative correlation with lactate levels (R = −0.484; *p* = 0.042).

## 4. Discussion

### 4.1. Key Findings

Statistically significant increases in WBC count, neutrophil count, and the neutrophil-to-lymphocyte ratio were observed in the postoperative period. These parameters, as shown in various studies, are well-established non-specific markers of systemic inflammatory response syndrome (SIRS) [12,13]. It appears that in the described case, the observed changes are related to a similar process. The etiology of this response is likely multifactorial, potentially attributable to surgical stress, exacerbation of underlying COPD, tissue injury, and intraoperative ventilatory stress.

From a clinical perspective, elevated postoperative inflammatory markers—particularly NLR—have been associated with increased risk of complications, prolonged hospitalization, and adverse outcomes in surgical patients, including those with COPD. Recognizing these changes may assist in the early identification of patients at higher risk of developing infectious or inflammatory complications in the perioperative period. This, in turn, may support more intensive monitoring, timely initiation of anti-inflammatory or antimicrobial therapy, and individualized postoperative care strategies. Further investigation is required to delineate the precise mechanisms contributing to this inflammatory response and to determine whether these laboratory changes may serve as reliable prognostic markers or therapeutic targets in perioperative management.

A notable decrease in hemoglobin, hematocrit, albumin, total protein, red blood cell counts, lymphocyte counts, eosinophil counts, and platelet counts was observed postoperatively. These changes are unlikely to be directly associated with COPD. Instead, they appear to reflect the physiological impact of the surgical procedure and the extent of intraoperative blood loss.

In this study, intraoperative ventilation parameters, including tidal volume, expired minute ventilation, positive end-expiratory pressure, mean airway pressure, peak airway pressure, and lung compliance, were not found to significantly impact hospital mortality, postoperative MV requirements, or MV duration. Furthermore, no associations were observed with the use of vasopressors or inotropes. However, it is challenging to conclude that intraoperative ventilation modes in patients with COPD are irrelevant to postoperative outcomes. These findings may reflect adjustments of respiratory parameters based on the baseline severity of patients’ conditions and comorbidities. Additionally, the limited data on respiratory support in the eICU database may have influenced the findings, underscoring the need for larger and more comprehensive datasets to derive more definitive conclusions.

An important finding from this study was the strong correlation between intraoperative plateau pressure and postoperative neutrophil-to-lymphocyte ratio. This observation supports the hypothesis that intraoperative respiratory patterns may influence outcomes in COPD patients. Further research is needed to explore and clarify this connection to better understand its implications for clinical management.

Similarly, the observed correlation between tidal volume and monocyte count may reflect underlying mechanistic links between mechanical ventilation and immune cell activation. It is known that excessive tidal volumes can lead to ventilator-induced lung injury and may contribute to systemic inflammation, which in turn could influence monocyte mobilization and differentiation. The association between plateau pressure and NLR—a recognized prognostic marker in inflammatory settings—also suggests that higher intraoperative airway pressures might contribute to immune dysregulation. These findings may point to subtle but clinically meaningful interactions between ventilation strategy and systemic immune response, particularly in patients with underlying pulmonary disease such as COPD. Recognizing these associations could inform future research aimed at optimizing lung-protective ventilation strategies in this population.

### 4.2. Relationship with Previous Studies

Doğan et al. investigated the prognostic ability of the SIRS score in predicting 1-month mortality in patients with Exacerbation of Chronic Obstructive Pulmonary Disease (AECOPD). Their findings revealed that the SIRS score had limited predictive value, with an area under the curve (AUC) of only 0.529 (95% CI: 0.3–1.1) [14]. In contrast, our study observed a statistically significant increase in WBC count, neutrophil count, and NLR during the postoperative period. This finding underscores the importance of exploring the potential impact of concomitant COPD on the progression of SIRS, particularly in postoperative settings.

The study by Futier et al. compared two intraoperative ventilation strategies in patients undergoing abdominal surgery, namely, traditional ventilation with tidal volumes of 10–12 mL/kg of predicted body weight without PEEP and protective ventilation with tidal volumes of 6–8 mL/kg combined with PEEP of 6–8 mbar and recruitment maneuvers. Their univariate analysis found that COPD was not associated with worse outcomes [15]. This finding aligns with the results of our study. However, it should be noted that in the cited study, only 10% of the 400 patients included in the cohort had COPD, which limits the generalizability of this conclusion.

It is worth noting, however, that the study by Futier et al. demonstrated an association between intraoperative ventilation parameters and postoperative outcomes [15]. In contrast, our analysis did not reveal such an association in patients with COPD. One possible explanation for this discrepancy lies in the population differences. In the study by Futier et al., only approximately 10% of patients had COPD, whereas our cohort was composed entirely of patients with moderate to severe COPD. As such, the underlying pathophysiology, disease burden, and ventilatory requirements in our population may differ substantially. Additionally, patients with COPD often undergo individualized ventilator adjustments based on their pre-existing pulmonary function and comorbidities, which could reduce the variability in ventilation strategies and potentially mask any measurable association with outcomes. Finally, differences in study design, sample size, statistical power, and data availability (particularly regarding detailed ventilator settings in our database) may also account for the observed divergence in findings. These factors underscore the need for further research specifically focused on COPD populations to clarify the role of intraoperative ventilation strategies in influencing postoperative outcomes.

Collectively, these studies suggest that intraoperative ventilation parameters and anesthetic approaches may significantly impact outcomes in patients with COPD. However, further investigations are warranted to substantiate these hypotheses and provide evidence-based guidelines for optimizing perioperative management in this high-risk group.

### 4.3. Significance of Study Findings

Although the discussed study did not demonstrate the impact of intraoperative ventilation patterns on the outcomes in COPD patients, it seems premature to consider this topic fully explored. The limited number of studies in this field, combined with the small sample sizes in the comparison groups, prevents us from drawing definitive conclusions or feeling reassured about the lack of impact of various intraoperative respiratory modes on morbidity and mortality in patients with COPD. However, this study highlights a significant gap in our understanding of the relationship between the chosen respiratory pattern and outcomes in COPD patients undergoing elective surgery.

### 4.4. Strengths and Limitations

Despite analyzing a database that includes a large number of patients and parameters, this study revealed that respiratory support parameters are reported very infrequently, with no information provided on mechanical ventilation modes. Additionally, the database did not include information on other potential confounding variables, such as the type of anesthesia, fluid management, hemodynamic status, and patient positioning. Furthermore, it is important to note that the data were collected from a cohort of patients whose treatment took place during the period of 2014–2015.

Since a correlation analysis was conducted, the following limitations must be acknowledged. Firstly, it is impossible to establish causality, as correlation analysis identifies associations between variables but does not determine whether one variable causes change in another. Secondly, the correlations may be influenced by third variables that were not accounted for in the analysis, potentially distorting the results. Thirdly, because correlation analysis assesses only linear relationships, non-linear associations may go undetected.

### 4.5. Future Studies and Prospects

Based on the results obtained in this study, there is a need for further randomized controlled trials aimed at identifying optimal respiratory support parameters both in the intraoperative and postoperative periods. Additionally, further investigations are required to identify predictors of mortality and potential exacerbation of COPD progression.

## 5. Conclusions

(1) A strong correlation was identified between intraoperative plateau pressure and the postoperative neutrophil-to-lymphocyte ratio, highlighting a potential link between intraoperative respiratory management and postoperative inflammatory responses.

(2) Intraoperative ventilation parameters, including tidal volume, expired minute ventilation, positive end-expiratory pressure, mean airway pressure, peak airway pressure, and lung compliance, were not found to significantly impact hospital mortality, postoperative MV requirements, or MV duration. Additionally, no associations were identified with the use of vasopressors or inotropes. Nevertheless, the obtained data must be interpreted in light of the existing limitations.

## Figures and Tables

**Table 1:** Initial parameters, surgery types, and comorbidities of patients with moderate or severe COPD.

Parameters		All Patients, N = 680	Moderate COPD, N = 535	Severe COPD, N = 145	*p*-Value
Sex	Male	361; 53%	274; 51%	87; 60%	0.060 ^1^
	Female	319; 47%	261; 49%	58; 40%	
Age, years		69 (61; 76)	69 (61; 77)	69 (61; 75)	0.704 ^2^
BMI, kg/m^2^		N = 660; 26.6 (22.5; 32.5)	N = 521; 26.7 (22.7; 32.3)	N = 139; 26.5 (21.7; 33.2)	0.816 ^2^
APACHE IV, score		N = 623; 51 (39; 67)	N = 494; 50 (38; 65)	N = 129; 53 (39.5; 70)	0.186 ^2^
Surgery types					
Abdominal surgery		226; 33%	173; 32%	53; 37%	0.339 ^1^
Cesarean section		1; 0.1%	1; 0.2%	0; 0%	>0.9 ^3^
Thrombectomy under general anesthesia		9; 1.3%	5; 0.9%	4; 2.8%	0.103 ^3^
Head and neck surgery		19; 3%	18; 3%	1; 0.7%	>0.9 ^3^
Mastectomy		2; 0.3%	2; 0.4%	0; 0%	>0.9 ^3^
Neurological surgery		48; 7%	41; 8%	7; 4.8%	0.237 ^1^
Orthopedic surgery		18; 3%	15; 3%	3; 2.1%	0.777 ^3^
Skin surgery		14; 2%	11; 2%	3; 2.1%	>0.9 ^3^
Thoracic surgery		149; 22%	106; 20%	43; 30%	0.011 ^1^
Trauma surgery		23; 3%	19; 4%	4; 2.8%	0.799 ^3^
Urogenital surgery		23; 3%	17; 3%	6; 4.1%	0.604 ^3^
Vascular surgery		132; 19%	115; 22%	17; 12%	0.008 ^1^
Other		16; 2%	12; 2%	4; 2.8%	0.757 ^3^
Comorbidities					
HIV		3; 0.4%	2; 0.4%	1; 0.7%	0.514 ^3^
AIDS		2; 0.3%	2; 0.4%	0; 0%	>0.9 ^3^
Anemia		1; 0.1%	0; 0%	1; 0.7%	0.213 ^3^
Angina		24; 4%	21; 4%	3; 2.1%	0.283 ^1^
Arrhythmia		81; 12%	59; 11%	22; 15%	0.172 ^1^
Arterial hypertension		412; 61%	335; 63%	77; 53%	0.038 ^1^
Coronary artery bypass grafting		46; 7%	35; 7%	11; 8%	0.657 ^1^
Procedural coronary intervention		64; 9%	50; 9%	14; 10%	>0.9 ^1^
Chronic heart failure		108; 16%	82; 15%	26; 18%	0.447 ^1^
Heart transplant		1; 0.1%	1; 0.2%	0; 0%	>0.9 ^3^
Heart valve disease		26; 4%	22; 4%	4; 2.8%	0.451 ^1^
Myocardial infarction		68; 10%	56; 11%	12; 8%	0.435 ^1^
Stroke		75; 11%	58; 11%	17; 12%	0.763 ^1^
Pulmonary embolism		15; 2%	9; 1.7%	6; 4.1%	0.104 ^3^
Deep vein thrombosis		29; 4%	19; 4%	10; 7%	0.077 ^1^
Peripheral vascular disease		77; 11%	63; 12%	14; 10%	0.475 ^1^
Oncology		114; 17%	93; 17%	21; 15%	0.407 ^1^
Lung cancer		48; 7%	43; 8%	5; 3.4%	0.056 ^1^
Respiratory failure		10; 2%	3; 0.6%	7; 4.8%	0.001 ^3^
Home oxygen		82; 12%	37; 7%	45; 31%	<0.001 ^1^
Asthma		98; 14%	80; 15%	18; 12%	0.440 ^1^
Restrictive pulmonary disease		12; 2%	7; 1.3%	5; 3.4%	0.144 ^3^
Lung transplant		1; 0.1%	0; 0%	1; 0.7%	0.213 ^3^
Chronic kidney disease		74; 11%	61; 11%	13; 9%	0.403 ^1^
Renal transplant		1; 0.1%	1; 0.2%	0; 0%	>0.9 ^3^
Liver cirrhosis		9; 1.3%	8; 1.5%	1; 0.7%	0.692 ^3^
Liver transplant		1; 0.1%	1; 0.2%	0; 0%	>0.9 ^3^
Peptic ulcer disease		22; 3%	19; 4%	3; 2.1%	0.595 ^3^
Hypothyroidism		58; 9%	44; 8%	14; 10%	0.584 ^1^
Insulin-dependent diabetes		80; 12%	64; 12%	16; 11%	0.758 ^1^
Sarcoidosis		2; 0.3%	1; 0.2%	1; 0.7%	0.381 ^3^
Neuromuscular disease		1; 0.1%	1; 0.2%	0; 0%	>0.9 ^3^
Seizures		28; 4%	25; 5%	3; 2.1%	0.162 ^1^

Categorical values are presented as numbers and frequencies; continuous values are presented as Me (IQR). ^1^—chi-square test; ^2^—Mann-Whitney U test; ^3^—Fisher’s exact test. Abbreviations: AIDS, acquired immunodeficiency syndrome; APACHE IV, Acute Physiology and Chronic Health Evaluation IV; BMI, body mass index; COPD, chronic obstructive pulmonary disease; HIV, human immunodeficiency virus; IQR, interquartile range.

**Table 2:** Pre- and postoperative laboratory parameters of patients admitted to the ICU after elective non-cardiac surgery.

Parameter	Preoperative	Postoperative	% of Change	*p*-Value (Pre-Post)
Glucose, mg/dL	N = 194; 116 (99; 141)	N = 643; 138 (114; 168)	N = 190; 12.3 (−8.7; 42.3)	<0.001
Potassium, mmol/L	N = 200; 4.1 (3.7; 4.4)	N = 654; 4.1 (3.8; 4.5)	N = 196; 2.4 (−9.3; 13.1)	0.288
Sodium, mmol/L	N = 199; 138 (135; 140)	N = 655; 138 (135; 140)	N = 195; 0.0 (−1.5; 2.2)	0.112
Hgb, g/dL	N = 198; 11.5 (9.8; 13.0)	N = 655; 10.9 (9.7; 12.3)	N = 193; −7.0 (−15.0; 0.9)	<0.001
Hct, %	N = 198; 35.5 (30.4; 39.9)	N = 656; 33.8 (29.5; 37.8)	N = 194; −7.1 (−14.8; 0.6)	<0.001
Creatinine, mg/dL	N = 200; 0.9 (0.7; 1.5)	N = 654; 0.9 (0.7; 1.3)	N = 196; −4.4 (−16.9; 12.5)	0.118
BUN, mg/dL	N = 200; 18.0 (11.3; 29.8)	N = 655; 17.0 (11.0; 24.0)	N = 196; 0.0 (−20.5; 21.3)	0.702
Platelets, K/mcL	N = 199; 232 (173; 310)	N = 650; 201 (156; 268)	N = 195; −3.9 (−16.7; 10.1)	0.002
WBC, K/mcL	N = 198; 9.7 (7.8; 13.7)	N = 648; 11.9 (8.8; 15.5)	N = 194; 19.5 (−6.5; 68.2)	<0.001
RBC, M/mcL	N = 198; 3.9 (3.4; 4.4)	N = 651; 3.7 (3.2; 4.2)	N = 194; −8.1 (−15.1; 0.3)	<0.001
Lymphs abs, K/mcL	N = 149; 1.2 (0.8; 1.8)	N = 487; 0.9 (0.6; 1.4)	N = 149; −28.1 (−58.1; 1.5)	<0.001
Polys abs, K/mcL	N = 139; 7.5 (4.8; 11.1)	N = 431; 10.0 (6.7; 13.4)	N = 123; 46.0 (−4.2; 101.1)	<0.001
NLR	N = 149; 5.9 (3.0; 11.9)	N = 484; 9.2 (5.1; 17.8)	N = 125; 64.8 (7.9; 211.6)	<0.001
Eos. abs, K/mcL	N = 150; 0.1 (0.0; 0.2)	N = 474; 0.0 (0.0; 0.1)	N = 90; −100.0 (−100.0; −30.3)	<0.001
Mon. abs, K/mcL	N = 149; 0.7 (0.5; 1.1)	N = 484; 0.7 (0.4; 1.1)	N = 133; −3.7 (−30.2; 56.6)	0.499
Baso abs, K/mcL	N = 141; 0.0 (0.0; 4.8)	N = 453; 0.0 (0.0; 2.3)	N = 58; −48.6 (−100.0; 25.1)	0.042
Albumin, g/dL	N = 133; 3.1 (2.4; 3.5)	N = 385; 2.6 (2.2; 3.1)	N = 106; −13.0 (−25.2; −3.5)	<0.001
AST, Units/L	N = 126; 20.0 (14.0; 28.3)	N = 355; 26.0 (17.0; 44.0)	N = 91; 4.3 (−18.9; 53.8)	0.228
ALT, Units/L	N = 125; 23.0 (13.5; 36.5)	N = 353; 21.0 (14.5; 36.0)	N = 91; −6.3 (−30.8; 20.0)	0.097
Total protein, g/dL	N = 126; 6.3 (5.6; 7.2)	N = 350; 5.4 (4.8; 6.0)	N = 92; −13.5 (−24.0; −5.4)	<0.001
Total bilirubin, mg/dL	N = 124; 0.6 (0.3; 0.8)	N = 352; 0.6 (0.4; 1.0)	N = 90; 31.0 (−20.0; 100.0)	0.002
Lactate, mmol/L	N = 41; 1.3 (1.0; 2.1)	N = 170; 1.6 (1.1; 2.6)	N = 21; 0.0 (−38.3; 97.2)	0.765
Troponin I, ng/mL	N = 24; 0.0 (0.0; 0.1)	N = 79; 0.1 (0.0; 0.2)	N = 9; 0.0 (−40.2; 200.0)	0.779
pH	N = 19; 7.4 (7.4; 7.4)	N = 332; 7.3 (7.3; 7.4)	N = 16; 0.1 (−1.3; 0.4)	0.798
CPK, Units/L	N = 13; 86.0 (46.0; 167.5)	N = 72; 178.0 (63.0; 529.0)	N = 4; 81.5 (−51.1; 223.6)	>0.9
BNP	N = 18; 502.5 (58.4; 1160.0)	N = 53; 560.0 (146.0; 1196.0)	N = 6; 22.1 (−4.5; 63.3)	0.249
PaCO_2_, mm Hg	N = 19; 41.6 (35.0; 49.3)	N = 337; 43.0 (37.7; 51.0)	N = 16; 7.6 (−11.0; 22.4)	0.605
O_2_ saturation, %	N = 10; 90.5 (87.6; 95.9)	N = 266; 97.6 (95.0; 99.0)	ND	0.012

Values are presented as Me (IQR). The assessment of laboratory parameters also included troponin T, fibrinogen, bands, and C-reactive protein (CRP), but these values were not presented due to missing data over 95%. Abbreviations: ALT, alanine aminotransferase; AST, aspartate aminotransferase; Baso abs, absolute basophil count; BNP, brain natriuretic peptide; BUN, blood urea nitrogen; CPK, creatine phosphokinase; CRP, C-reactive protein, Eos abs, absolute eosinophil count; Hct, hematocrit; Hgb, hemoglobin; ICU, intensive care unit; Lymphs abs, absolute lymphocyte count; Mon. abs, absolute monocyte count; ND, no data; NLR, neutrophil-to-lymphocyte ratio; PaCO_2_, partial pressure of carbon dioxide; Polys abs, absolute neutrophil count; RBC, red blood cell count; WBC, white blood cell count.

**Table 3:** Impact of intraoperative ventilation parameters on the studied outcomes.

Outcomes	Hospital Mortality			ICU Mortality			Duration of Mechanical Ventilation			Use of Mechanical Ventilation			Use of Vasopressors and Inotropes		
Ventilation Parameters	Alive, N = 621	Expired, N = 59	*p*-Value	Alive, N = 650	Expired, N = 30	*p*-Value	1 Day, N = 91	>1 Days, N = 189	*p*-Value	Yes (MV), N = 280	No (Spont), N = 400	*p*-Value	Yes, N = 86	No, N = 594	*p*-Value
PEEP, cm H_2_O	N = 64; 5 (5; 5)	N = 16; 5 (5; 5)	0.844	N = 73; 5 (5; 5)	N = 7; 5 (5; 5)	0.452	N = 30; 5 (5; 8)	N = 37; 5 (5; 5)	0.404	N = 67; 5 (5; 5)	N = 13; 5 (5; 5)	0.372	N = 15; 5 (5; 5)	N = 65; 5 (5; 5)	0.893
TV, mL/kg	N = 58; 8.0 (7.3; 10.0)	N = 12; 7.5 (7.3; 8.2)	0.115	N = 64; 7.9 (7.3; 9.8)	N = 6; 7.5 (7.2; 8.4)	0.288	N = 24; 7.8 (7.1; 8.8)	N = 34; 7.9 (7.3; 10.2)	0.622	N = 58; 7.9 (7.3; 9.9)	N = 12; 7.9 (7.4; 9.5)	0.945	N = 15; 7.6 (7.0; 8.8)	N = 55; 8.0 (7.3; 9.9)	0.200
P_mean_, cm H_2_O	N = 38; 9.0 (8.0; 11.0)	N = 12; 9.7 (8.1; 12.0)	0.537	N = 45; 9.7 (8.0; 11.5)	N = 5; 8.5 (7.0; 9.2)	0.115	N = 9; 9.1 (8.4; 11.0)	N = 32; 9.6 (8.0; 12.0)	0.889	N = 41; 9.4 (8.0; 11.5)	N = 9; 9.0 (7.3; 10.4)	0.383	N = 14; 9.6 (8.4; 10.5)	N = 36; 9.0 (8.0; 11.0)	0.693
P_peak_, cm H_2_O	N = 28; 24.0 (19.0; 26.8)	N = 10; 22.5 (19.5; 25.8)	0.961	N = 33; 24.0 (19.5; 27.0)	N = 5; 21.0 (16.0; 24.5)	0.310	N = 6; 20.0 (18.3; 22.0)	N = 23; 25.0 (19.0; 28.0)	0.278	N = 29; 24.0 (19.0; 27.5)	N = 9; 24.0 (17.5; 25.6)	0.866	N = 12; 25.0 (21.8; 30.0)	N = 26; 22.0 (18.8; 26.0)	0.129
EMV, L/min	N = 33; 7.2 (6.0; 8.9)	N = 11; 7.7 (7.0; 10.1)	0.334	N = 39; 7.3 (6.0; 9.4)	N = 5; 7.6 (4.5; 11.2)	0.886	N = 9; 7.3 (6.5; 9.7)	N = 26; 7.5 (6.0; 9.7)	0.810	N = 35; 7.4 (6.0; 9.6)	N = 9; 6.7 (5.4; 9.6)	0.474	N = 12; 7.6 (6.1; 9.3)	N = 32; 7.3 (6.0; 9.8)	0.948
SaO_2_, %	N = 5; 96.0 (92.0; 98.5)	ND	<0.001	N = 5; 96.0 (92.0; 98.5)	ND	<0.001	N = 2; 98.5 (98.0; 0.0)	ND	<0.001	N = 2; 98.5 (98.0; 0.0)	N = 3; 94.0 (90.0; 0.0)	0.200	ND	N = 5; 96.0 (92.0; 98.5)	<0.001
P_plateau_, cm H_2_O	N = 23; 18.0 (13.0; 20.0)	N = 5; 17.0 (12.5; 19.5)	0.6	N = 26; 18.5 (13.8; 20.0)	N = 2; 12.5 (12.0; 0.0)	0.106	N = 7; 19.0 (13.0; 20.0)	N = 14; 16.5 (12.8; 20.0)	0.585	N = 21; 17.0 (13.0; 20.0)	N = 7; 19.0 (14.0; 21.0)	0.376	N = 5; 13.0 (11.5; 15.0)	N = 23; 19.0 (15.0; 20.0)	0.016
Compliance, mL/cm H_2_O	N = 11; 66.0 (40.0; 88.0)	N = 5; 43.2 (21.6; 51.0)	0.115	N = 14; 47.5 (39.9; 83.6)	N = 2; 44.5 (31.0; 0.0)	0.5	N = 4; 41.6 (39.6; 76.8)	N = 7; 58.0 (31.0; 91.0)	0.648	N = 11; 44.0 (39.4; 88.0)	N = 5; 51.0 (37.7; 67.5)	0.913	N = 5; 58.0 (28.1; 80.0)	N = 11; 43.2 (39.4; 82.1)	0.743
FiO_2_, %	N = 110; 50.0 (40.0; 65.0)	N = 21; 60.0 (50.0; 100.0)	0.015	N = 120; 50.0 (40.0; 77.5)	N = 11; 80.0 (50.0; 100.0)	0.012	N = 34; 55.0 (40.0; 100.0)	N = 59; 50.0 (40.0; 99.0)	0.482	N = 93; 50.0 (40.0; 100.0)	N = 38; 40.0 (40.0; 60.0)	0.008	N = 28; 50.0 (42.5; 75.0)	N = 103; 50.0 (40.0; 100.0)	0.598

Values are presented as Me (IQR). The analysis of the impact of the intraoperative parameters on the studied outcomes also included peak flow and continuous positive airway pressure (CPAP), but the results were not presented due to missing data over 95%. Abbreviations: EMV, expired minute ventilation; FiO_2_, the fraction of inspired oxygen; IQR, interquartile range; MV, mechanical ventilation; ND, no data; PEEP, positive end-expiratory pressure; P_peak_, peak inspiratory pressure; P_mean_, mean airway pressure; P_plateau_, plateau pressure; SaO_2_, arterial oxygen saturation; Spont, spontaneous breathing; TV, tidal volume. The comparison of complications incidence by intraoperative ventilation parameters was not possible due to the small number of observed cases (Table S1).

**Table 4:** Correlations between ventilation parameters and outcomes: hospital LoS and ICU LoS.

Outcomes	Hospital LoS 6.6 (IQR 3.2 to 10.5)		ICU LoS 1.9 (IQR 1.0 to 3.6)	
Ventilation Parameters	R [95% CI]	*p*-Value	R [95% CI]	*p*-Value
PEEP, N = 80	0.142 [−0.087; 0.357]	0.208	−0.076 [−0.296; 0.153]	0.505
Tidal volume, N = 70	−0.23 [−0.447; 0.013]	0.056	−0.036 [−0.276; 0.207]	0.765
P_mean_, N = 50	0.373 [0.097; 0.595]	0.008	0.115 [−0.177; 0.389]	0.426
P_peak_, N = 38	0.223 [−0.114; 0.514]	0.179	0.193 [−0.144; 0.49]	0.245
EMV, N = 44	0.098 [−0.213; 0.392]	0.526	−0.012 [−0.316; 0.294]	0.936
SaO_2_, N = 5	0.7 [−0.508; 0.98]	0.188	0.600 [−0.625; 0.972]	0.285
P_plateau_, N = 28	0.217 [−0.181; 0.554]	0.266	−0.042 [−0.419; 0.346]	0.831
Compliance, N = 16	0.241 [−0.304; 0.667]	0.368	0.006 [−0.503; 0.512]	0.983
Peak flow, N = 10	−0.4 [−0.83; 0.326]	0.252	−0.113 [−0.704; 0.571]	0.757
FiO_2_, N = 131	0.08 [−0.097; 0.253]	0.361	0.086 [−0.092; 0.258]	0.331

Abbreviations: EMV, expired minute ventilation; FiO_2_, the fraction of inspired oxygen; ICU, intensive care unit; IQR, interquartile range; LoS, length of stay; PEEP, positive end-expiratory pressure; P_peak_, peak inspiratory pressure; P_mean_, mean airway pressure; P_plateau_, plateau pressure; SaO_2_, arterial oxygen saturation.

## Data Availability

The original contributions presented in this study are included in the article/Supplementary Materials; further inquiries can be directed to the corresponding author.

## Supplementary Materials

The following supporting information can be downloaded at https://www.mdpi.com/article/10.3390/jcm14072438/s1, Supplemental S1: STROBE Statement, checklist of items that should be included in reports of cohort studies; Table S1: Impact of intraoperative ventilation parameters on the incidence of studied complications Table S2: Spearman’s correlation between ventilation parameters, postoperative laboratory parameters, and percentage of change between pre- and postoperative values.

## References

[1] The Top 10 Causes of Death. https://www.who.int/news-room/fact-sheets/detail/the-top-10-causes-of-death.

[2] Wang H., Ye X., Zhang Y., Ling S. (2022). Global, Regional, and National Burden of Chronic Obstructive Pulmonary Disease from 1990 to 2019. Front. Physiol..

[3] Li H.-Y., Gao T.-Y., Fang W., Xian-Yu C.-Y., Deng N.-J., Zhang C., Niu Y.-M. (2023). Global, Regional and National Burden of Chronic Obstructive Pulmonary Disease over a 30-Year Period: Estimates from the 1990 to 2019 Global Burden of Disease Study. Respirology.

[4] AL Wachami N., Guennouni M., Iderdar Y., Boumendil K., Arraji M., Mourajid Y., Bouchachi F.Z., Barkaoui M., Louerdi M.L., Hilali A. (2024). Estimating the Global Prevalence of Chronic Obstructive Pulmonary Disease (COPD): A Systematic Review and Meta-Analysis. BMC Public Health.

[5] Gupta H., Ramanan B., Gupta P.K., Fang X., Polich A., Modrykamien A., Schuller D., Morrow L.E. (2013). Impact of COPD on Postoperative Outcomes: Results from a National Database. Chest.

[6] Osadnik C.R., Tee V.S., Carson-Chahhoud K.V., Picot J., Wedzicha J.A., Smith B.J. (2017). Non-Invasive Ventilation for the Management of Acute Hypercapnic Respiratory Failure Due to Exacerbation of Chronic Obstructive Pulmonary Disease. Cochrane Database Syst. Rev..

[7] Khrapov K.N., Kovalev M.G., Sedov S.S. (2020). Preparation for Anesthesia of Patients with Concomitant Lung Pathology and a High Risk of Developing Postoperative Pulmonary Complications. Messenger Anesthesiol. Resusc..

[8] Zabolotskikh I.B., Gritsan A.I., Kirov M.Y., Kuzovlev A.N., Lebedinskii K.M., Mazurok V.A., Protsenko D.N., Trembach N.V., Shadrin R.V., Yaroshetskiy A.I. (2022). Perioperative Management of Patients with Respiratory Failure: Methodological Recommendations of the All-Russian Public Organization “Federation of Anesthesiologists and Reanimatologists”. Ann. Crit. Care.

[9] Mirza S., Clay R.D., Koslow M.A., Scanlon P.D. (2018). COPD Guidelines: A Review of the 2018 GOLD Report. Mayo Clin. Proc..

[10] Pollard T.J., Johnson A.E.W., Raffa J.D., Celi L.A., Mark R.G., Badawi O. (2018). The EICU Collaborative Research Database, a Freely Available Multi-Center Database for Critical Care Research. Sci. Data.

[11] Mukaka M.M. (2012). A Guide to Appropriate Use of Correlation Coefficient in Medical Research. Malawi Med. J..

[12] Sikora J.P., Karawani J., Sobczak J. (2023). Neutrophils and the Systemic Inflammatory Response Syndrome (SIRS). Int. J. Mol. Sci..

[13] Kriplani A., Pandit S., Chawla A., de la Rosette J.J.M.C.H., Laguna P., Jayadeva Reddy S., Somani B.K. (2022). Neutrophil-Lymphocyte Ratio (NLR), Platelet-Lymphocyte Ratio (PLR) and Lymphocyte-Monocyte Ratio (LMR) in Predicting Systemic Inflammatory Response Syndrome (SIRS) and Sepsis after Percutaneous Nephrolithotomy (PNL). Urolithiasis.

[14] Doğan N.Ö., Özturan İ.U., Pekdemir M., Yaka E., Yılmaz S. (2024). Prognostic Value of Early Warning Scores in Patients Presenting to the Emergency Department with Exacerbation of COPD. Med. Klin. Intensivmed. Notfallmedizin.

[15] Futier E., Constantin J.-M., Paugam-Burtz C., Pascal J., Eurin M., Neuschwander A., Marret E., Beaussier M., Gutton C., Lefrant J.-Y. (2013). A Trial of Intraoperative Low-Tidal-Volume Ventilation in Abdominal Surgery. N. Engl. J. Med..

