# Supplementary material for: The Impact of Intraoperative Respiratory Patterns on Morbidity and Mortality in Patients with COPD Undergoing Elective Surgery
Source: J Clin Med. 2025 Apr 3;14(7):2438. doi: 10.3390/jcm14072438 (PMC11989855; doi:10.3390/jcm14072438)
Supplement: Supplementary file 1 [file jcm-14-02438-s001.zip › jcm-3540373-supplementary.pdf]

Supplementary Material

1. Supplemental S1 – STROBE Statement, checklist of items that should be included in reports of *cohort studies*

	Item No	Recommendation	Location
**Title and abstract**	1	(*a*) Indicate the study's design with a commonly used term in the title or the abstract	Abstract
		(*b*) Provide in the abstract an informative and balanced summary of what was done and what was found	Abstract
**Introduction**			
Background/rationale	2	Explain the scientific background and rationale for the investigation being reported	Introduction
Objectives	3	State specific objectives, including any prespecified hypotheses	Introduction
**Methods**			
Study design	4	Present key elements of study design early in the paper	Selection criteria
Setting	5	Describe the setting, locations, and relevant dates, including periods of recruitment, exposure, follow-up, and data collection	Data sources
Participants	6	(*a*) Give the eligibility criteria, and the sources and methods of selection of participants. Describe methods of follow-up	Data sources; Selection criteria
		(*b*) For matched studies, give matching criteria and number of exposed and unexposed	NA
Variables	7	Clearly define all outcomes, exposures, predictors, potential confounders, and effect modifiers. Give diagnostic criteria, if applicable	Outcomes
Data sources/ measurement	8*	For each variable of interest, give sources of data and details of methods of assessment (measurement). Describe comparability of assessment methods if there is more than one group	Data sources; Outcomes
Bias	9	Describe any efforts to address potential sources of bias	Data sources
Study size	10	Explain how the study size was arrived at	NA (whole database analysis)
Quantitative variables	11	Explain how quantitative variables were handled in the analyses. If applicable, describe which groupings were chosen and why	Outcomes
Statistical methods	12	(*a*) Describe all statistical methods, including those used to control for confounding	Statistical analysis
		(*b*) Describe any methods used to examine subgroups and interactions	Statistical analysis
		(*c*) Explain how missing data were addressed	Statistical analysis
		(*d*) If applicable, explain how loss to follow-up was addressed	NA
		(*e*) Describe any sensitivity analyses	Statistical analysis
**Results**			
Participants	13*	(a) Report numbers of individuals at each stage of study—eg numbers potentially eligible, examined for eligibility, confirmed eligible, included in the study, completing follow-up, and analysed	Figure 1

		(b) Give reasons for non-participation at each stage	Figure 1
		(c) Consider use of a flow diagram	Figure 1
Descriptive data	14*	(a) Give characteristics of study participants (eg demographic, clinical, social) and information on exposures and potential confounders	Table 1
		(b) Indicate number of participants with missing data for each variable of interest	Table 1
		(c) Summarise follow-up time (eg, average and total amount)	NA
Outcome data	15*	Report numbers of outcome events or summary measures over time	Table 1
Main results	16	(*a*) Give unadjusted estimates and, if applicable, confounder-adjusted estimates and their precision (eg, 95% confidence interval). Make clear which confounders were adjusted for and why they were included	Table 2; Table 3; Table 4; Figure 2; Table S1; Table S2
		(*b*) Report category boundaries when continuous variables were categorized	Table 3
		(*c*) If relevant, consider translating estimates of relative risk into absolute risk for a meaningful time period	NA
Other analyses	17	Report other analyses done—eg analyses of subgroups and interactions, and sensitivity analyses	NA
**Discussion**			
Key results	18	Summarise key results with reference to study objectives	Key findings
Limitations	19	Discuss limitations of the study, taking into account sources of potential bias or imprecision. Discuss both direction and magnitude of any potential bias	Strengths and limitations
Interpretation	20	Give a cautious overall interpretation of results considering objectives, limitations, multiplicity of analyses, results from similar studies, and other relevant evidence	Conclusion
Generalisability	21	Discuss the generalisability (external validity) of the study results	Significance of study findings
**Other information**			
Funding	22	Give the source of funding and the role of the funders for the present study and, if applicable, for the original study on which the present article is based	Meta data

*Give information separately for exposed and unexposed groups.

Note: An Explanation and Elaboration article discusses each checklist item and gives methodological background and published examples of transparent reporting. The STROBE checklist is best used in conjunction with this article (freely available on the Web sites of PLoS Medicine at http://www.plosmedicine.org/, Annals of Internal Medicine at http://www.annals.org/, and Epidemiology at http://www.epidem.com/). Information on the STROBE Initiative is available at http://www.strobe-statement.org.

2. Table S1. Impact of intraoperative ventilation parameters on the incidence of studied complications.

**Outcomes**	*Heart failure*			*Acute kidney injury*			*Respiratory failure*			*Arrhythmia*			*Congestive heart failure*			*Septic shock*		
**Ventilation parameters**	*Yes, N=16*	*No, N=664*	*p-value*	*Yes, N=33*	*No, N=647*	*p-value*	*Yes, N=44*	*No, N=636*	*p-value*	*Yes, N=41*	*No, N=639*	*p-value*	*Yes, N=13*	*No, N=667*	*p-value*	*Yes, N=15*	*No, N=665*	*p-value*
PEEP, cm H_2O	N = 4; 5.0 (3.5; 5.0)	N = 76; 5.0 (5.0; 5.0)	0.205	N = 5; 5.0 (5.0; 7.5)	N = 75; 5.0 (5.0; 5.0)	0.878	N = 8; 5.0 (5.0; 5.0)	N = 72; 5.0 (5.0; 5.0)	0.179	N = 4; 5.0 (3.5; 5.0)	N = 76; 5.0 (5.0; 5.0)	0.205	N = 3; 5.0 (3.0; ND)	N = 77; 5.0 (5.0; 5.0)	0.199	N = 3; 5.0 (5.0; 5.0)	N = 77; 5.0 (5.0; 5.0)	0.648
TV, mL/kg	N = 4; 8.9 (6.6; 10.4)	N = 66; 7.9 (7.3; 9.6)	0.836	N = 5; 6.9 (6.2; 10.8)	N = 65; 7.9 (7.3; 9.8)	0.337	N = 9; 7.8 (7.2; 10.0)	N = 61; 7.9 (7.3; 9.6)	0.850	N = 3; 7.8 (7.3; ND)	N = 67; 7.9 (7.3; 9.6)	0.892	N = 3; 10.0 (7.8; ND)	N = 67; 7.9 (7.3; 9.5)	0.225	N = 3; 7.0 (6.9; ND)	N = 67; 7.9 (7.3; 9.9)	0.067
P_{mean}, cm H_2O	N = 2; 10.9 (9.8; ND)	N = 48; 9.0 (8.0; 11.0)	0.367	N = 5; 10.0 (9.6; 16.5)	N = 45; 9.0 (8.0; 11.0)	0.059	N = 8; 9.9 (8.7; 11.8)	N = 42; 9.0 (8.0; 11.0)	0.328	N = 3; 11.0 (8.0; ND)	N = 47; 9.0 (8.0; 11.0)	0.409	**N = 1; 12.0 (12.0; 12.0)**	**N = 49; 9.0 (8.0; 11.0)**	**<0.001**	N = 2; 9.0 (8.6; ND)	N = 48; 9.1 (8.0; 11.0)	0.754
P_{peak}, cm H_2O	**N = 1; 24.0 (24.0; 24.0)**	**N = 37; 24.0 (19.0; 26.5)**	**<0.001**	N = 3; 30.0 (24.0; ND)	N = 35; 23.0 (19.0; 26.0)	0.065	N = 4; 26.0 (24.3; 36.8)	N = 34; 23.0 (18.8; 26.0)	0.113	**N = 1; 25.0 (25.0; 25.0)**	**N = 37; 24.0 (19.0; 26.5)**	**<0.001**	ND	N = 38; 24.0 (19.0; 26.3)	NA	ND	N = 38; 24.0 (19.0; 26.3)	NA
EMV, L/min	**N = 1; 8.4 (8.4; 8.4)**	**N = 43; 7.3 (6.0; 9.6)**	**<0.001**	N = 4; 6.9 (4.8; 8.1)	N = 40; 7.5 (6.0; 9.8)	0.395	N = 5; 6.7 (6.0; 289.2)	N = 39; 7.4 (6.0; 9.6)	0.971	N = 2; 287.4 (4.8; ND)	N = 42; 7.4 (6.0; 9.5)	0.888	ND	N = 44; 7.4 (6.0; 9.6)	NA	**N = 1; 4.2 (4.2; 4.2)**	**N = 43; 7.4 (6.0; 9.6)**	**<0.001**
SaO_2, %	ND	N = 5; 96.0 (92.0; 98.5)	NA	ND	N = 5; 96.0 (92.0; 98.5)	NA	ND	N = 5; 96.0 (92.0; 98.5)	NA	ND	N = 5; 96.0 (92.0; 98.5)	NA	ND	N = 5; 96.0 (92.0; 98.5)	NA	ND	N = 5; 96.0 (92.0; 98.5)	NA
$P_{plateau}$, cm H_2O	N = 3; 13.0 (13.0; ND)	N = 25; 18.0 (13.5; 20.0)	0.351	N = 2; 17.0 (13.0; ND)	N = 26; 17.5 (13.0; 20.0)	0.894	N = 3; 13.0 (13.0; ND)	N = 25; 18.0 (13.5; 20.0)	0.673	N = 2; 16.0 (13.0; ND)	N = 26; 17.5 (13.0; 20.0)	0.698	N = 2; 16.0 (13.0; ND)	N = 26; 17.5 (13.0; 20.0)	0.698	ND	N = 28; 17.5 (13.0; 20.0)	NA
Compliance, mL/cm H_2O	**N = 1; 69.0 (69.0; 69.0)**	**N = 15; 44.0 (39.4; 82.1)**	**<0.001**	**N = 1; 69.0 (69.0; 69.0)**	**N = 15; 44.0 (39.4; 82.1)**	**<0.001**	N = 2; 50.0 (31.0; ND)	N = 14; 47.5 (39.9; 83.6)	0.700	ND	N = 16; 47.5 (39.6; 78.8)	NA	ND	N = 16; 47.5 (39.6; 78.8)	NA	ND	N = 16; 47.5 (39.6; 78.8)	NA
Peak Flow, L/min	**N = 1; 65.0 (65.0; 65.0)**	**N = 9; 60.0 (36.2; 60.0)**	**<0.001**	**N = 1; 65.0 (65.0; 65.0)**	**N = 9; 60.0 (36.2; 60.0)**	**<0.001**	**N = 1; 65.0 (65.0; 65.0)**	**N = 9; 60.0 (36.2; 60.0)**	**<0.001**	ND	N = 10; 60.0 (36.3; 61.3)	NA	ND	N = 10; 60.0 (36.3; 61.3)	NA	ND	N = 10; 60.0 (36.3; 61.3)	NA
CPAP, cm H_2O	**N = 1; 5 (5; 5)**	**N = 9; 5 (5; 5)**	**<0.001**	N = 2; 5 (5; 5)	N = 8; 5 (5; 5)	NA	N = 4; 5 (5; 5)	N = 6; 5 (5; 5)	NA	**N = 1; 5 (5; 5)**	**N = 9; 5 (5; 5)**	**<0.001**	ND	N = 10; 5 (5; 5)	NA	ND	N = 10; 5 (5; 5)	NA

FiO_2, %	N = 6; 80.0 (47.5; 100.0)	N = 125; 50.0 (40.0; 86.5)	0.145	N = 6; 60.0 (44.5; 85.0)	N = 125; 50.0 (40.0; 98.5)	0.565	N = 9; 60.0 (50.0; 100.0)	N = 122; 50.0 (40.0; 94.3)	0.103	N = 8; 60.0 (40.0; 100.0)	N = 123; 50.0 (40.0; 93.0)	0.580	N = 5; 100.0 (50.0; 100.0)	N = 126; 50.0 (40.0; 83.3)	0.102	N = 4; 50.0 (33.5; 72.5)	N = 127; 50.0 (40.0; 99.0)	0.619

Values are presented as Me (IQR).

Abbreviations: *CPAP, continuous positive airway pressure; EMV, exhaled minute volume; FiO_2, fraction of inspired oxygen; IQR, interquartile range; NA, not applicable; ND, no data; PEEP, positive end-expiratory pressure; P_{peak}, peak inspiratory pressure; P_{mean}, mean airway pressure; $P_{plateau}$, plateau pressure; SaO_2, arterial oxygen saturation; TV, tidal volume.*

3. Table S2. Spearman's correlation between ventilation parameters, postoperative laboratory parameters and percentage of change between pre- and postoperative values.

Laboratory parameters	Type of value	*PEEP, cm H_2O*	*TV, mL/ kg*	*P_{mean}, cm H_2O*	*P_{peak}, cm H_2O*	*EMV, L/min*	*SaO_2, %*	*$P_{plateau}$, cm H_2O*	*Compliance, mL/cm H_2O*	*Peak flow, L/min*	*FiO_2, %*
Glucose, mg/dL	R	0.032	0.127	0.115	0.201	0.159	0.500	0.267	-0.335	0.144	0.006
	p	0.778	0.301	0.442	0.248	0.321	0.391	0.197	0.263	0.692	0.947
	N	78	68	47	35	41	5	25	13	10	125
Glucose, % of change	R	-0.095	-0.173	-0.043	0.199	-0.013	1.000	0.334	-0.500	NA	-0.025
	p	0.632	0.430	0.864	0.514	0.965	NA	0.518	0.667	NA	0.879
	N	28	23	18	13	15	2	6	3	3	40
Potassium, mmol/L	R	-0.066	0.032	0.076	0.099	0.165	0.872	-0.074	**0.580**	0.148	-0.049
	p	0.565	0.794	0.607	0.567	0.296	0.054	0.718	**0.030**	0.684	0.584
	N	79	68	48	36	42	5	26	14	10	127
Potassium, % of change	R	0.098	0.156	0.424	0.517	-0.299	1.000	0.638	-1.000	NA	-0.165
	p	0.619	0.477	0.079	0.070	0.279	NA	0.173	NA	NA	0.301
	N	28	23	18	13	15	2	6	3	3	41
Sodium, mmol/L	R	0.009	0.101	0.018	0.099	0.030	0.300	0.041	**-0.558**	0.147	0.101
	p	0.936	0.415	0.902	0.564	0.850	0.624	0.842	**0.038**	0.684	0.260
	N	79	68	48	36	42	5	26	14	10	127
Sodium, % of change	R	-0.237	0.076	-0.233	-0.072	-0.010	1.000	-0.213	0.500	NA	0.090
	p	0.225	0.732	0.353	0.815	0.972	NA	0.686	0.667	NA	0.580
	N	28	23	18	13	15	2	6	3	3	40
Hgb, g/dL	R	-0.147	-0.110	-0.193	-0.072	**-0.461**	0.100	-0.105	-0.207	0.110	-0.159
	p	0.195	0.371	0.190	0.677	**0.002**	0.873	0.611	0.478	0.762	0.071
	N	79	68	48	36	42	5	26	14	10	129
Hgb, % of change	R	0.012	0.139	-0.082	0.277	-0.402	-1.000	-0.698	-1.000	NA	0.042
	p	0.953	0.526	0.747	0.360	0.137	NA	0.123	NA	NA	0.795
	N	28	23	18	13	15	2	6	3	3	41
Hct, %	R	-0.159	-0.093	-0.192	-0.029	**-0.425**	0.300	-0.075	-0.222	0.181	**-0.177**
	p	0.162	0.449	0.190	0.868	**0.005**	0.624	0.717	0.446	0.616	**0.045**
	N	79	68	48	36	42	5	26	14	10	129
Hct, % of change	R	0.093	0.040	-0.160	0.241	-0.468	-1.000	-0.698	-1.000	NA	-0.020
	p	0.637	0.858	0.525	0.428	0.078	NA	0.123	NA	NA	0.902
	N	28	23	18	13	15	2	6	3	3	41
Creatinine, mg/dL	R	-0.095	**-0.280**	**0.370**	**0.375**	0.114	**0.900**	0.120	0.156	0.319	-0.054
	p	0.404	**0.021**	**0.010**	**0.024**	0.473	**0.037**	0.558	0.594	0.369	0.550
	N	79	68	48	36	42	5	26	14	10	127

Laboratory parameters	Type of value	*PEEP, cm H_2O*	*TV, mL/ kg*	*P_{mean}, cm H_2O*	*P_{peak}, cm H_2O*	*EMV, L/min*	*SaO_2, %*	*$P_{plateau}$, cm H_2O*	*Compliance, mL/cm H_2O*	*Peak flow, L/min*	*FiO_2, %*
Creatinine, % of change	R	-0.284	0.098	-0.025	-0.365	0.278	1.000	0.030	-0.500	NA	-0.258
	p	0.143	0.657	0.920	0.220	0.315	NA	0.954	0.667	NA	0.104
	N	28	23	18	13	15	2	6	3	3	41
BUN, mg/dL	R	0.064	**-0.414**	**0.332**	0.169	0.281	0.300	-0.148	0.134	**0.675**	0.061
	p	0.576	**<0.001**	**0.021**	0.325	0.071	0.624	0.472	0.647	**0.032**	0.498
	N	79	68	48	36	42	5	26	14	10	127
BUN, % of change	R	-0.118	-0.155	0.330	0.124	0.209	1.000	-0.152	-0.500	NA	-0.017
	p	0.551	0.480	0.181	0.685	0.454	NA	0.774	0.667	NA	0.916
	N	28	23	18	13	15	2	6	3	3	41
Platelets, K/mcL	R	0.150	0.058	0.050	0.164	-0.134	0.600	0.010	0.442	0.000	-0.097
	p	0.188	0.637	0.735	0.340	0.398	0.400	0.961	0.114	1.000	0.280
	N	79	68	48	36	42	4	26	14	10	127
Platelets, % of change	R	0.054	**-0.446**	**0.638**	0.396	0.270	-1.000	0.334	-0.500	NA	0.263
	p	0.785	**0.033**	**0.004**	0.181	0.331	NA	0.518	0.667	NA	0.096
	N	28	23	18	13	15	2	6	3	3	41
WBC, K/mcL	R	-0.038	**0.286**	0.005	0.076	0.012	0.600	0.091	0.284	0.232	-0.004
	p	0.738	**0.018**	0.975	0.658	0.942	0.400	0.657	0.326	0.519	0.965
	N	79	68	48	36	42	4	26	14	10	125
WBC, % of change	R	-0.172	0.309	0.179	-0.036	0.148	-1.000	-0.334	1.000	NA	0.056
	p	0.382	0.151	0.477	0.907	0.598	NA	0.518	NA	NA	0.730
	N	28	23	18	13	15	2	6	3	3	41
RBC, M/mcL	R	-0.149	-0.062	-0.193	-0.060	**-0.321**	1.000	-0.138	-0.183	0.188	-0.152
	p	0.189	0.613	0.188	0.728	**0.038**	NA	0.502	0.532	0.604	0.087
	N	79	68	48	36	42	4	26	14	10	127
RBC, % of change	R	0.018	0.004	-0.163	0.241	-0.386	-1.000	-0.698	-1.000	NA	0.102
	p	0.928	0.986	0.519	0.428	0.155	NA	0.123	NA	NA	0.525
	N	28	23	18	13	15	2	6	3	3	41
Lymphs abs, K/mcL	R	-0.101	**0.327**	-0.143	-0.079	0.079	0.200	-0.237	0.233	0.676	0.072
	p	0.441	**0.017**	0.399	0.689	0.671	0.800	0.343	0.546	0.140	0.474
	N	60	53	37	28	31	4	18	9	6	100
Lymphs abs, % of change	R	-0.172	0.227	0.402	0.193	0.469	-1.000	-0.738	NA	NA	-0.008
	p	0.496	0.381	0.195	0.647	0.203	NA	0.262	NA	NA	0.967
	N	18	17	12	8	9	2	4	1	3	28
Polys abs, K/mcL	R	0.053	**0.332**	0.064	0.286	-0.222	0.500	0.353	0.700	-0.169	-0.092
	p	0.708	**0.020**	0.735	0.208	0.298	0.667	0.197	0.188	0.749	0.392
	N	52	49	30	21	24	3	15	5	6	89
	R	-0.143	0.039	0.062	0.058	-0.214	-1.000	-0.866	NA	NA	0.137

Laboratory parameters	Type of value	PEEP, cm H_2O	TV, mL/ kg	P_{mean}, cm H_2O	P_{peak}, cm H_2O	EMV, L/min	SaO_2, %	$P_{plateau}$, cm H_2O	Compliance, mL/cm H_2O	Peak flow, L/min	FiO_2, %
Polys abs, % of change	p	0.583	0.889	0.866	0.913	0.645	NA	0.333	NA	NA	0.503
	N	17	15	10	6	7	2	3	ND	3	26
NLR	R	0.023	-0.069	0.191	0.301	-0.086	-0.200	**0.708**	-0.322	-0.507	-0.119
	p	0.859	0.625	0.257	0.119	0.644	0.800	**0.001**	0.398	0.305	0.242
	N	60	53	37	28	31	4	18	9	6	99
NLR, % of change	R	0.112	-0.143	-0.006	-0.203	-0.429	1.000	0.000	NA	NA	0.076
	p	0.668	0.612	0.987	0.700	0.337	NA	1.000	NA	NA	0.711
	N	17	15	10	6	7	2	3	ND	3	26
Eos. abs, K/mcL	R	-0.211	0.061	-0.033	0.157	0.136	-0.500	0.130	-0.655	0.000	-0.067
	p	0.118	0.670	0.854	0.464	0.500	0.667	0.632	0.158	1.000	0.520
	N	56	52	34	24	27	3	16	6	6	95
Eos. abs, % of change	R	-0.261	-0.256	0.151	0.335	0.213	-1.000	NA	NA	NA	-0.494
	p	0.466	0.579	0.747	0.581	0.686	NA	NA	NA	NA	0.072
	N	10	7	7	5	6	2	1	ND	3	14
Mon. abs, K/mcL	R	-0.061	0.266	0.039	0.317	0.189	1.000	0.025	-0.543	**0.845**	-0.038
	p	0.652	0.056	0.826	0.123	0.336	NA	0.927	0.266	**0.034**	0.711
	N	57	52	34	25	28	4	16	6	6	97
Mon. abs, % of change	R	-0.051	**0.611**	-0.025	-0.029	-0.429	-1.000	-0.866	NA	NA	-0.059
	p	0.846	**0.016**	0.946	0.957	0.337	NA	0.333	NA	NA	0.775
	N	17	15	10	6	7	2	3	ND	3	26
Baso abs, K/mcL	R	-0.125	0.148	0.018	0.392	-0.357	0.866	0.044	-0.304	0.000	-0.078
	p	0.384	0.321	0.921	0.058	0.067	0.333	0.887	0.558	1.000	0.471
	N	51	47	34	24	27	3	13	6	6	87
Baso abs, % of change	R	NA	0.000	0.462	0.738	0.500	NA	NA	NA	NA	-0.647
	p	NA	1.000	0.434	0.262	0.391	NA	NA	NA	NA	0.165
	N	3	4	5	4	5	ND	1	ND	1	6
Albumin, g/dL	R	-0.024	-0.095	-0.005	0.242	0.258	NA	0.383	-0.598	0.264	-0.030
	p	0.864	0.527	0.977	0.223	0.141	NA	0.087	0.068	0.568	0.795
	N	52	47	39	27	34	1	21	10	7	79
Albumin, % of change	R	0.234	-0.364	0.501	0.487	0.619	NA	1.000	NA	NA	0.411
	p	0.464	0.245	0.116	0.268	0.102	NA	NA	NA	NA	0.114
	N	12	12	11	7	8	ND	2	1	1	16
AST, Units/L	R	-0.029	0.058	-0.081	0.111	-0.271	NA	0.125	-0.212	-0.600	0.094
	p	0.841	0.709	0.627	0.590	0.128	NA	0.600	0.556	0.208	0.424
	N	49	44	38	26	33	ND	20	10	6	74
AST, % of change	R	-0.135	**0.915**	-0.268	-0.700	0.143	NA	1.000	NA	NA	-0.156
	p	0.710	**<0.001**	0.486	0.188	0.787	NA	NA	NA	NA	0.593

Laboratory parameters	Type of value	*PEEP, cm H_2O*	*TV, mL/ kg*	*P_{mean}, cm H_2O*	*P_{peak}, cm H_2O*	*EMV, L/min*	*SaO_2, %*	*$P_{plateau}$, cm H_2O*	*Compliance, mL/cm H_2O*	*Peak flow, L/min*	*FiO_2, %*
	N	10	10	9	5	6	ND	2	1	ND	14
ALT, Units/L	R	-0.075	-0.021	-0.046	0.000	-0.200	NA	0.219	-0.067	-0.429	0.220
	p	0.610	0.890	0.782	1.000	0.263	NA	0.353	0.855	0.397	0.059
	N	49	44	38	26	33	ND	20	10	6	74
ALT, % of change	R	0.202	0.479	0.067	-0.200	0.200	NA	1.000	NA	NA	-0.196
	p	0.575	0.162	0.864	0.747	0.704	NA	NA	NA	NA	0.503
	N	10	10	9	5	6	ND	2	1	ND	14
Total protein, g/dL	R	0.194	-0.282	0.143	0.034	**0.347**	NA	0.275	-0.128	0.000	**-0.240**
	p	0.186	0.067	0.390	0.868	**0.048**	NA	0.241	0.724	1.000	**0.042**
	N	48	43	38	26	33	ND	20	10	6	72
Total protein, % of change	R	0.315	-0.055	0.628	0.100	**0.886**	NA	1.000	NA	NA	0.426
	p	0.376	0.881	0.070	0.873	**0.019**	NA	NA	NA	NA	0.129
	N	10	10	9	5	6	ND	2	1	ND	14
Total bilirubin, mg/dL	R	-0.051	-0.290	-0.126	-0.068	-0.074	NA	0.049	**-0.815**	0.257	0.035
	p	0.726	0.056	0.450	0.740	0.681	NA	0.836	**0.004**	0.623	0.771
	N	49	44	38	26	33	ND	20	10	6	73
Total bilirubin, % of change	R	0.157	-0.067	-0.092	0.300	-0.086	NA	-1.000	NA	NA	-0.257
	p	0.664	0.855	0.814	0.624	0.872	NA	NA	NA	NA	0.375
	N	10	10	9	5	6	ND	2	1	ND	14
Lactate, mmol/L	R	-0.247	0.072	0.004	**-0.484**	0.333	NA	-0.636	0.500	0.348	**0.327**
	p	0.187	0.701	0.986	**0.042**	0.130	NA	0.090	0.391	0.499	**0.019**
	N	30	31	28	18	22	ND	8	5	6	51
Lactate, % of change	R	-0.577	0.771	**-0.900**	-0.866	-1.000	NA	NA	NA	NA	-0.147
	p	0.308	0.072	**0.037**	0.333	NA	NA	NA	NA	NA	0.781
	N	5	6	5	3	3	ND	ND	ND	1	6
Troponin I, ng/mL	R	NA	-0.314	-0.192	0.564	-0.543	NA	-0.154	1.000	0.500	-0.026
	p	NA	0.346	0.649	0.322	0.266	NA	0.805	NA	0.667	0.916
	N	12	11	8	5	6	ND	5	2	3	19
pH	R	-0.131	0.106	0.037	-0.025	0.237	-1.000	-0.334	0.533	-0.013	0.026
	p	0.337	0.445	0.824	0.898	0.178	NA	0.176	0.139	0.972	0.816
	N	56	54	38	28	34	2	18	9	10	80
pH, % of change	R	**-0.949**	0.400	-1.000	-1.000	0.500	NA	NA	NA	NA	0.127
	p	**0.014**	0.600	NA	NA	0.667	NA	NA	NA	NA	0.786
	N	5	4	3	3	3	ND	ND	ND	1	7
CPK, Units/L	R	-0.114	0.103	-0.566	**-0.928**	-0.690	NA	-0.335	NA	NA	-0.073
	p	0.754	0.777	0.112	**0.008**	0.058	NA	0.581	NA	NA	0.788
	N	10	10	9	6	8	ND	5	ND	1	16

Laboratory parameters	Type of value	***PEEP, cm H_2O***	***TV, mL/ kg***	***P_{mean}, cm H_2O***	***P_{peak}, cm H_2O***	***EMV, L/min***	***SaO_2, %***	***$P_{plateau}$, cm H_2O***	***Compliance, mL/cm H_2O***	***Peak flow, L/min***	***FiO_2, %***
Bands, %	R	0.321	-0.127	-0.205	-0.700	0.000	NA	-0.289	0.500	NA	0.165
	p	0.243	0.695	0.741	0.188	1.000	NA	0.637	0.667	NA	0.453
	N	15	12	5	5	5	1	5	3	1	23
BNP, pg/mL	R	0.218	0.429	-0.200	0.400	-0.051	NA	-0.218	-1.000	1.000	-0.437
	p	0.604	0.397	0.747	0.600	0.935	NA	0.638	NA	NA	0.206
	N	8	6	5	4	5	ND	7	2	2	10
Troponin T, ng/mL	R	-0.500	1.000	NA	NA	NA	NA	NA	NA	NA	-0.500
	p	0.667	NA	NA	NA	NA	NA	NA	NA	NA	0.391
	N	3	2	1	1	1	ND	1	ND	1	5
Fibrinogen, mg/dL	R	0.131	-0.500	0.500	NA	-1.000	NA	-1.000	NA	NA	-0.289
	p	0.805	0.391	0.667	NA	NA	NA	NA	NA	NA	0.487
	N	6	5	3	1	2	ND	2	1	1	8
CRP, mg/dL	R	0.707	-0.200	0.316	NA	-0.400	NA	-0.600	NA	-1.000	0.224
	p	0.182	0.800	0.684	NA	0.600	NA	0.400	NA	NA	0.718
	N	5	4	4	1	4	ND	4	ND	2	5

R, Spearman's correlation coefficient; p, p-value; N, number of cases.

The analysis also included assessment of correlation between ventilation parameters and percent of change (post- vs preoperative) of troponin I and T, creatine phosphokinase, bands, brain natriuretic peptide, fibrinogen, and C-reactive peptide, but these values were not presented due to lack of data.

The coefficients of the statistically significant correlations are highlighted with yellow colour. The brightest are for those with p-value < 0.001.

Abbreviations: *ALT, alanine aminotransferase; AST, aspartate aminotransferase; Baso abs, absolute basophil count; BUN, blood urea nitrogen; EMV, exhaled minute volume; Eos abs, absolute eosinophil count; FiO_2, fraction of inspired oxygen; Hct, hematocrit; Hgb, hemoglobin; Lymphs abs, absolute lymphocyte count; Mon. abs, absolute monocyte count; NA, not applicable; ND, no data; NLR, neutrophil-to-lymphocyte ratio; PEEP, positive end-expiratory pressure; P_{peak}, peak inspiratory pressure; P_{mean}, mean airway pressure; Polys abs, absolute neutrophil count; $P_{plateau}$, plateau pressure; RBC, red blood cell count; SaO_2, arterial oxygen saturation; TV, tidal volume; WBC, white blood cell count.*
